# Combination of pemetrexed with bevacizumab for non-small-cell lung cancer: a meta-analysis study

**DOI:** 10.1186/s13019-024-02975-6

**Published:** 2024-08-02

**Authors:** Wei Fang, Xingqiao Peng, Qun Zhou

**Affiliations:** 1 Department of Respiratory, Nan’an District People’s Hospital, Chongqing, 400060 China; 2 Comprehensive Cancer Center, Daping Hospital, Army Medical University, No. 10 Daping Changjiang branch Road, Yuzhong District, Chongqing, 400042 China

**Keywords:** NSCLC, Pemetrexed, Bevacizumab, Overall survival, Progression-free survival

## Abstract

**Background:**

Combining pemetrexed with bevacizumab may have some potential in improving the efficacy in patients with non-small-cell lung cancer (NSCLC), and this meta-analysis aims to explore the impact of pemetrexed addition to bevacizumab on treatment efficacy for NSCLC.

**Methods:**

PubMed, EMbase, Web of science, EBSCO, and Cochrane library databases were systematically searched, and we included randomized controlled trials (RCTs) assessing the effect of pemetrexed addition to bevacizumab on treatment efficacy in patients with NSCLC. Overall survival and progression-free survival were included in this meta-analysis.

**Results:**

Four RCTs were finally included in the meta-analysis. Overall, compared with bevacizumab for NSCLC, pemetrexed addition showed significantly improved overall survival (hazard ratio [HR] = 0.87; 95% confidence interval [CI] = 0.76 to 0.99; *P* = 0.03), survival rate (odd ratio [OR] = 1.41; 95% CI = 1.06 to 1.86; *P* = 0.02), progression-free survival (HR = 0.63; 95% CI = 0.55 to 0.72; *P* < 0.00001) and progression-free survival rate (OR = 1.92; 95% CI = 1.38 to 2.67; *P* < 0.00001), but led to the increase in grade ≥ 3 adverse events (OR = 2.15; 95% CI = 1.62 to 2.84; *P* < 0.00001).

**Conclusions:**

Pemetrexed addition may be effective to improve treatment efficacy for NSCLC compared to bevacizumab treatment.

## Introduction

Molecular-targeted anticancer drugs and immune checkpoint inhibitors (ICIs) were commonly used to improve the outcomes of patients with non-small-cell lung cancer (NSCLC) [1–5], while platinum-based chemotherapy was one key therapeutic option for NSCLC without epidermal growth factor receptor (EGFR) mutation [6–10]. Especially, bevacizumab and pemetrexed displayed an important role in treating NSCLC [11–14].

In the subgroup analysis of one phase III study, cisplatin and pemetrexed resulted in a significant improvement in overall survival (OS) compared to cisplatin and gemcitabine in patients with advanced NSCLC [15]. In the JMEN trial, maintenance therapy with pemetrexed supplementation significantly prolonged OS and progression-free survival (PFS) in patients with NSCLC without disease progression [16]. These suggested that maintenance therapy with pemetrexed may be a promising option for patients with NSCLC.

Several RCTs showed that pemetrexed addition to bevacizumab may have the capability to improve the outcomes for patients with NSCLC, but the results were not well established [17–19]. We therefore conducted this meta-analysis of RCTs to evaluate the effectiveness of pemetrexed addition to bevacizumab on treatment efficacy for NSCLC.

## Materials and methods

### Study selection and data collection

This meta-analysis was conducted by using previously studies, so ethical approval and patient consent were not needed. It was conducted according to the Preferred Reporting Items for Systematic Reviews and Meta-analysis statement and Cochrane Handbook for Systematic Reviews of Interventions [20, 21].

We have searched PubMed, EMbase, Web of science, EBSCO and the Cochrane library up to September 2023, by using the search terms “pemetrexed” AND “bevacizumab” AND “lung cancer” OR “NSCLC”. The inclusion criteria were as follows: (1) study design was RCT; (2) patients were diagnosed with NSCLC; (3) intervention treatments were pemetrexed plus bevacizumab versus bevacizumab. Patients with uncontrolled hypertension, major hemoptysis within 4 weeks, recent major surgery within 6 weeks, significant cardiovascular disease, and cavitary lung lesions were excluded.

### Assessment for risk of bias

The risk of bias tool was used to assess the quality of individual studies in accordance with the *Cochrane Handbook for Systematic Reviews of Interventions* [22], and the following sources of bias were considered: selection bias, performance bias, attrition bias, detection bias, reporting bias, and other potential sources of bias. The overall risk of bias for each study was evaluated and rated: low, unclear, and high [23]. Two investigators independently searched articles, extracted data, and assessed the quality of included studies. Any discrepancy was solved by consensus.

### Outcome measures

The following information was extracted: first author, publication year, sample size, age, weight, body mass index, adenocarcinoma and methods of two groups. The primary outcomes were overall survival and survival rate. Secondary outcomes included progression-free survival, progression-free survival rate and grade ≥ 3 adverse events.

### Statistical analysis

A team consisting of three authors did the statistical analyses. Hazard ratio (HR) with 95% confidence interval [CI] was used to assess continuous outcomes and odd ratio (OR) with 95% CI was used to assess dichotomous outcomes. I^2^ statistic was used to assess the heterogeneity, and significant heterogeneity was observed when *I*^*2*^ > 50% [24, 25]. The random-effect model was used regardless of the heterogeneity. We conducted the sensitivity analysis through detecting the influence of a single study on the overall estimate via omitting one study in turn or using the subgroup analysis. *P* < 0.05 indicated statistical significance and Review Manager Version 5.3 was used in all statistical analyses.

### Quality of evidence

The quality of evidence for each outcome was evaluated based on the methodological quality and the confidence in the results, and it was assessed by GRADE recommendations as high quality, moderate quality, low quality, or very low quality [26].

## Results

### Literature search, study characteristics and quality assessment

**Fig. 1:**
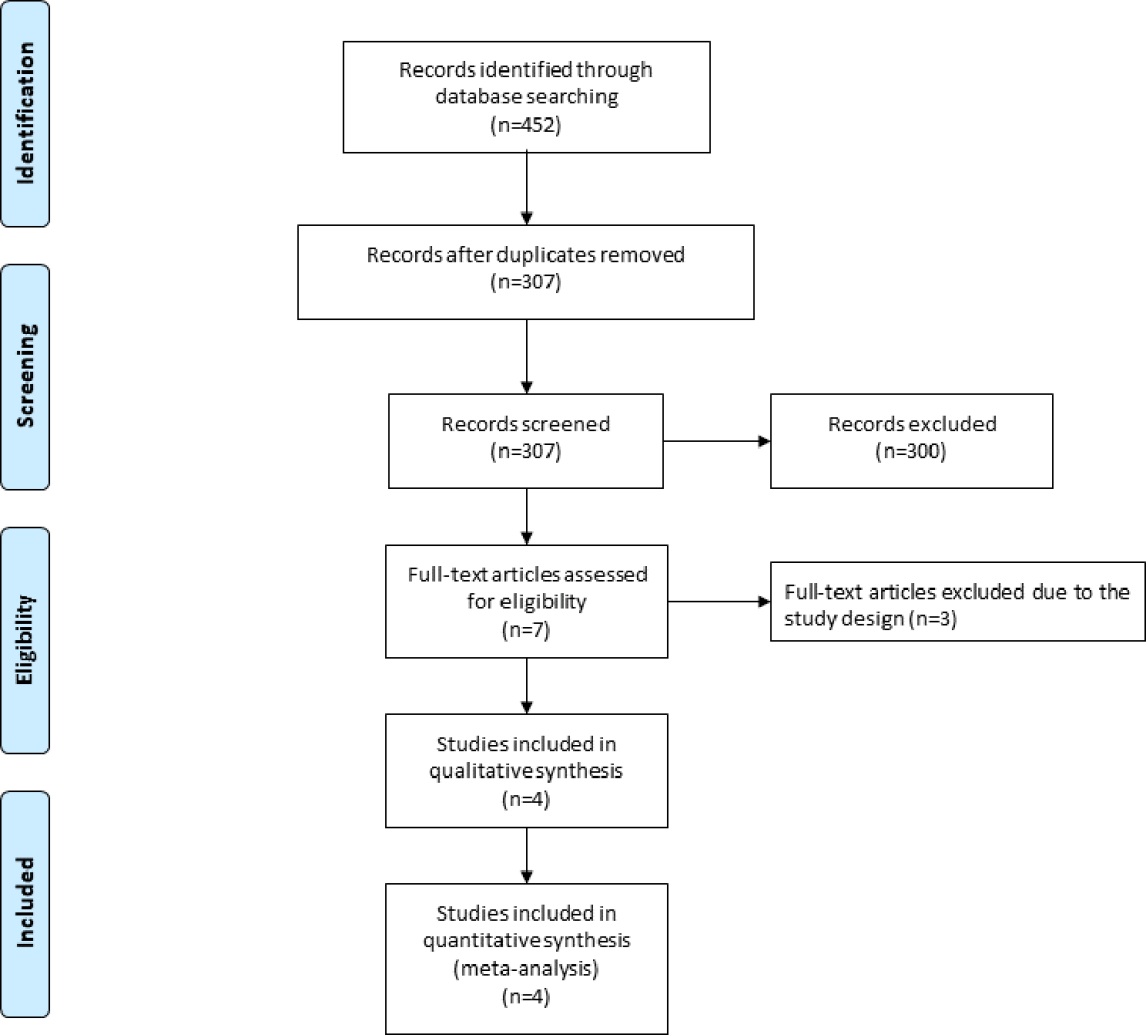
Flow diagram of study searching and selection process

The flow chart for the selection process and detailed identification was presented in Fig. 1. 452 publications were searched after the initial search of databases. 145 duplicates and 301 papers after checking the titles/abstracts were excluded. Three studies were removed because of the study design. Ultimately, four RCTs were included in the meta-analysis [17–19, 27].

The baseline characteristics of four eligible RCTs in the meta-analysis were summarized in Table 1. The four studies were published between 2013 and 2020, and total sample size was 1467. There were similar baseline characteristics between pemetrexed group and control group. The treatment duration of pemetrexed addition ranged from 8 to 63 months. The methods of chemotherapies included bevacizumab 7.5 mg/kg or/and pemetrexed 500 mg/m2 once every 3 weeks (Table 2).

**Table 1:** Characteristics of included studies

NO.	Author	Pemetrexed group					Control group					
		Number	Age (years)	Male (*n*)	Tumor histologic subtype (adenocarcinoma)	Methods	Number	Age (years)	Male (*n*)	Tumor histologic subtype (adenocarcinoma)	Methods	Median follow-up time
1	Yoshida [17]	21	67(39–73), median (range)	14	19	bevacizumab (15 mg/kg) plus pemetrexed (500 mg/m2) every three weeks	22	66 (40–74)	14	21	bevacizumab (15 mg/kg) every 3 weeks	31.6 years
2	Seto [18]	301	65 (32–77), median (range)	221	289	pemetrexed 500 mg/m2, and bevacizumab 15 mg/kg once every 3 weeks for 4 cycles	298	65 (27–81), median (range)	209	284	bevacizumab 15 mg/kg once every 3 weeks for 4 cycles	63.3 months
3	Ramalingam [19]	293	64, median	143	268	bevacizumab (15 mg/kg) and pemetrexed (500 mg/m 2) every 3 weeks	287	65	40	261	bevacizumab (15 mg/kg) every 3 weeks	50.6 months
4	Barlesi [27]	125	< 65 (88)	72	107	bevacizumab 7.5 mg/kg plus pemetrexed 500 mg/m2 once every 3 weeks	120	< 65 (85)	68	110	bevacizumab 7.5 mg/kg every 3 weeks	8.1 months

**Table 2:**
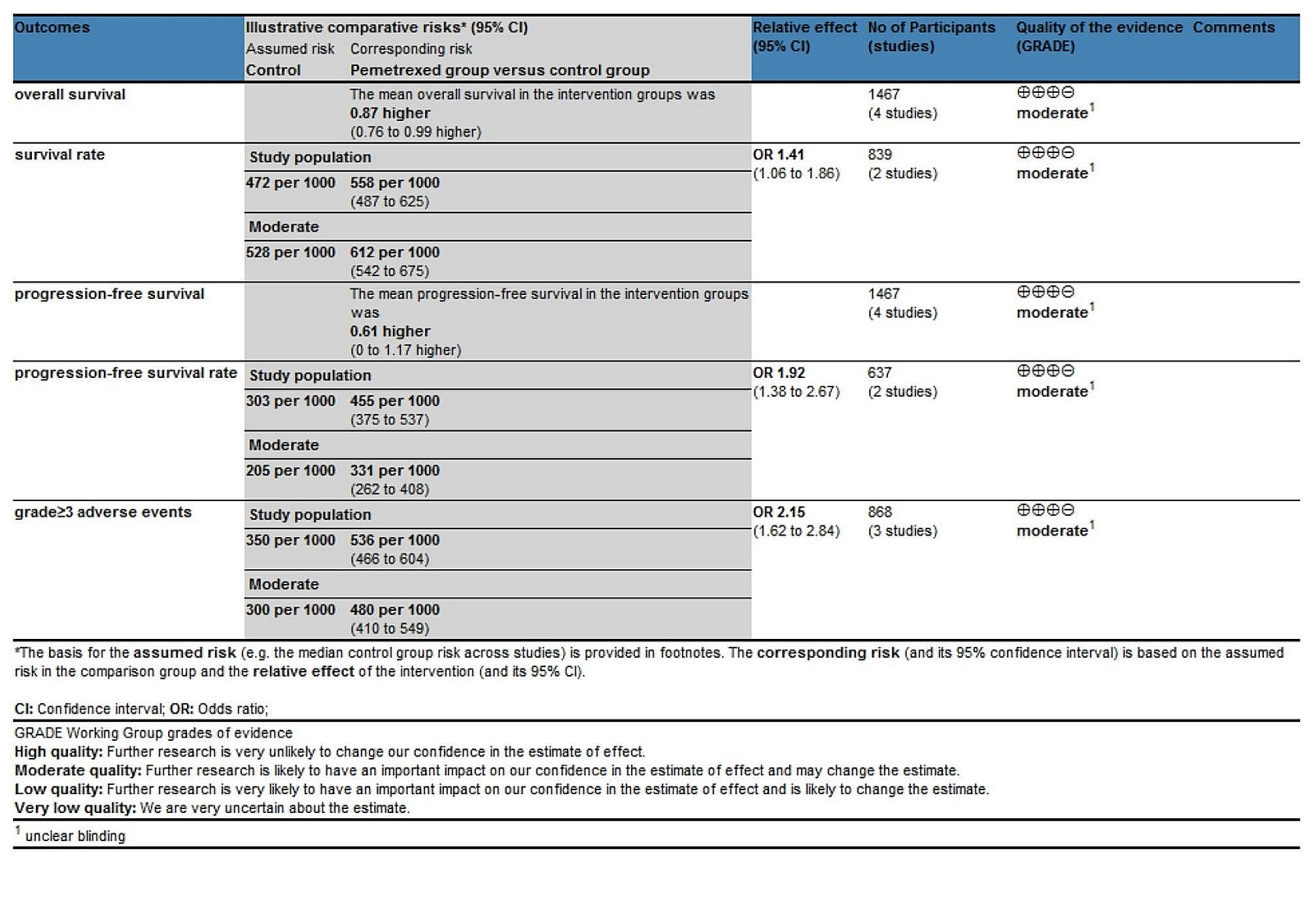
The quality of evidence for each outcome by GRADE recommendations

**Fig. 2:**
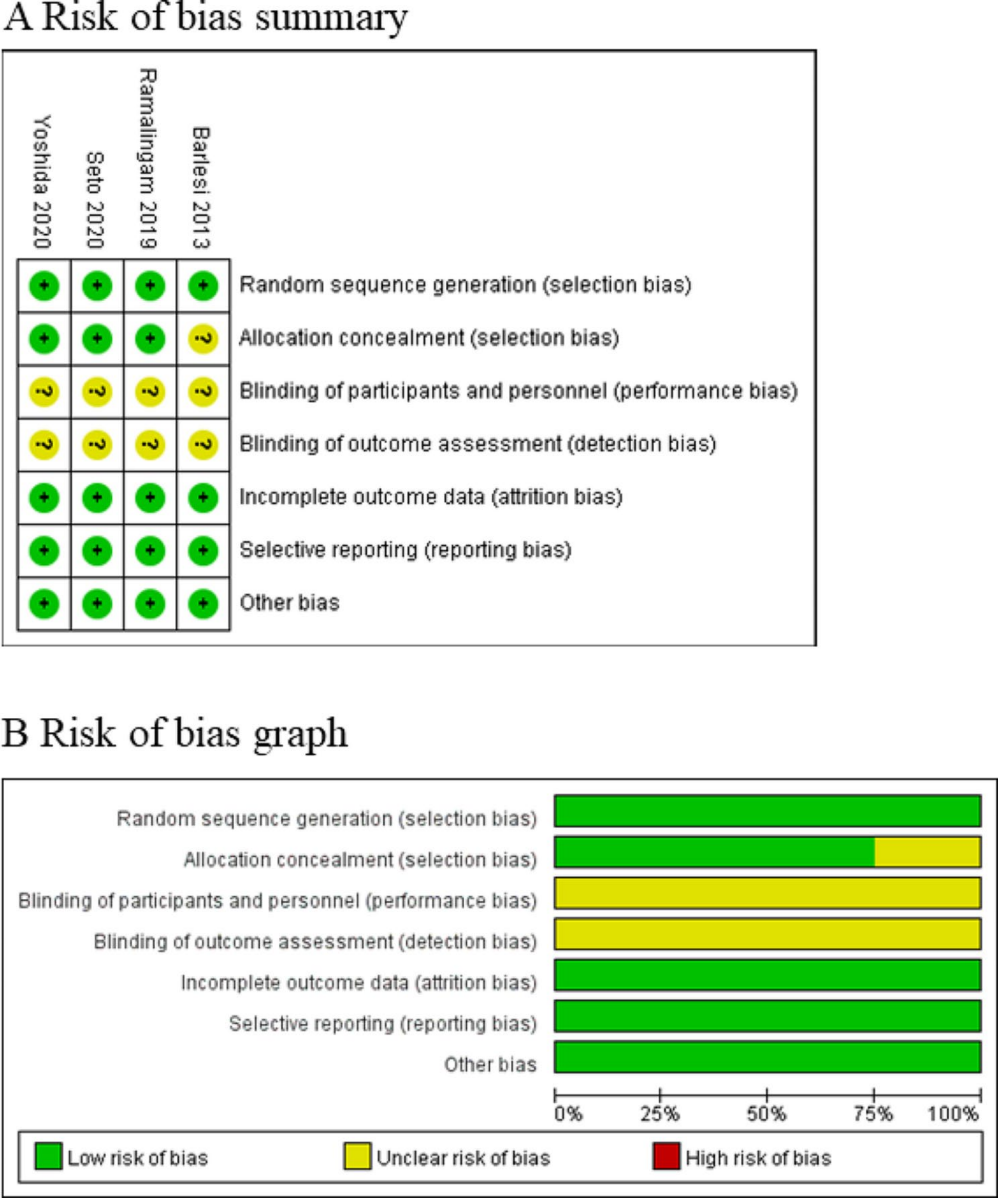
Risk of bias assessment. (**A**) Authors’ judgments about each risk of bias item for each included study. (**B**) Authors’ judgments about each risk of bias item presented as percentages across all included studies

Among the four RCTs, four studies reported overall survival [17–19, 27], two studies reported survival rate [18, 27], four studies reported progression-free survival [17–19, 27], two studies reported progression-free survival rate [17, 18], and three studies reported grade ≥ 3 adverse events [17, 19, 27]. Risk of bias analysis showed that four studies had unclear risk of performance bias and detection bias [17–19, 27], while one study showed unclear risk of selection bias (Fig. 2) [27]. However, all four RCTs generally had high quality.

### Primary outcomes: overall survival and survival rate

**Fig. 3:**
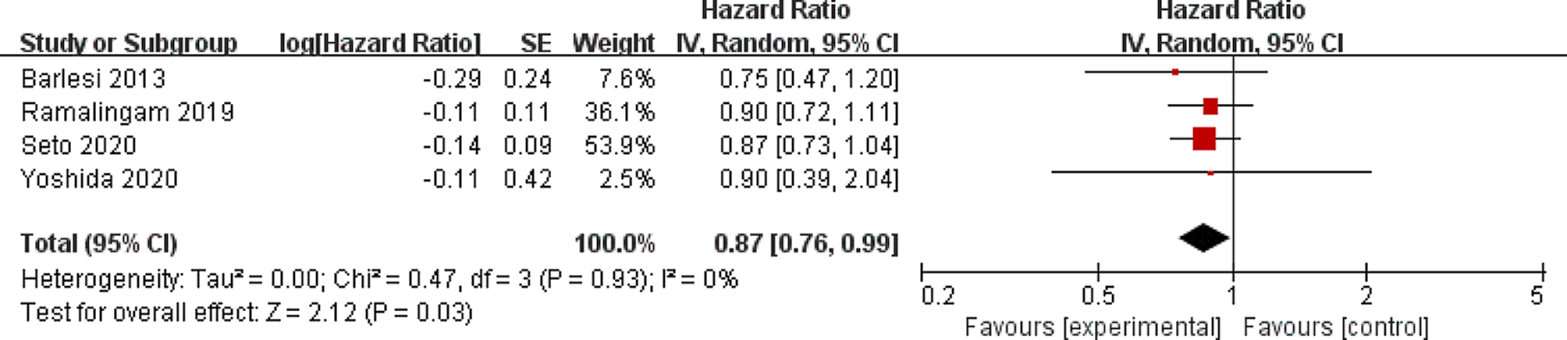
Forest plot for the meta-analysis of overall survival

**Fig. 4:**
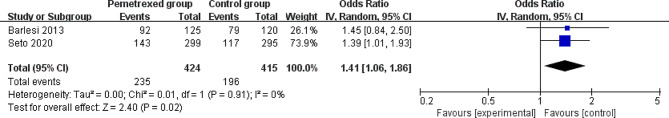
Forest plot for the meta-analysis of survival rate

Compared to control group for NSCLC, pemetrexed addition was associated with significantly prolonged overall survival (moderate quality, HR = 0.87; 95% CI = 0.76 to 0.99; *P* = 0.03) with no heterogeneity among the studies (I^2^ = 0%, heterogeneity *P* = 0.93, Fig. 3) and increased survival rate (moderate quality, OR = 1.41; 95% CI = 1.06 to 1.86; *P* = 0.02) with no heterogeneity among the studies (I^2^ = 0%, heterogeneity *P* = 0.91, Fig. 4).

### Sensitivity analysis

**Fig. 5:**
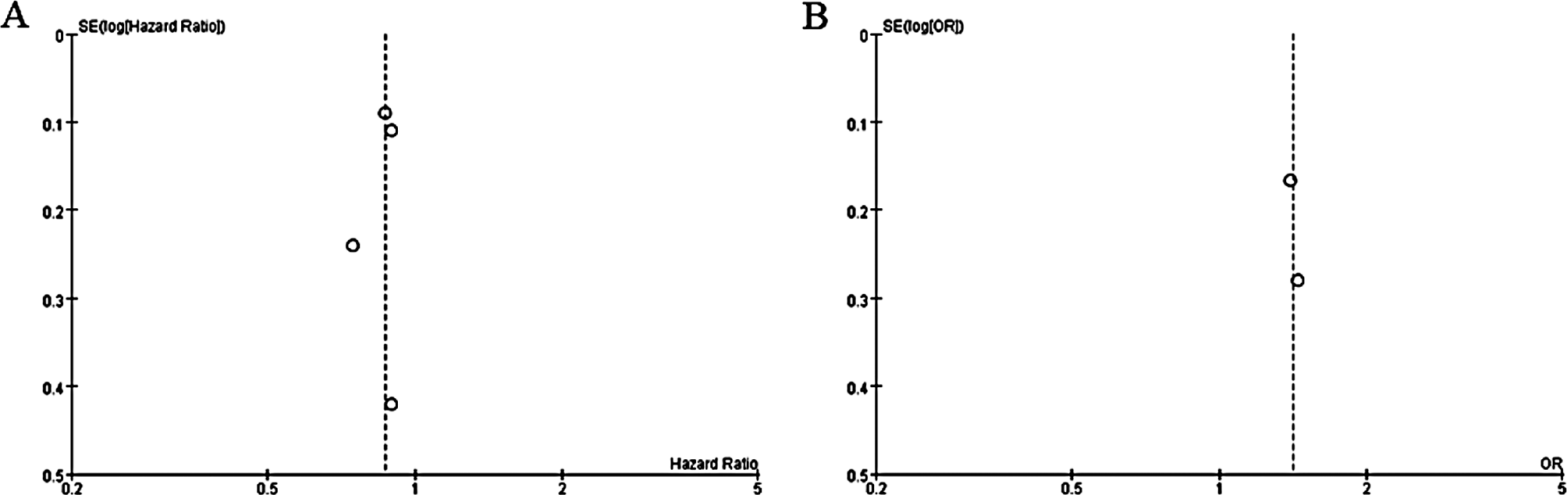
Funnel plot for the meta-analysis of overall survival (**A**) and survival rate (**B**)

No heterogeneity was observed for the primary outcomes, and thus we did not perform the sensitivity analysis by omitting one study in turn for the meta-analysis. The funnel plot was relatively symmetrical for overall survival (Fig. 5A) and survival rate (Fig. 5B), and all studies almost fell within the 95% CI axis. There was little evidence of publication bias.

### Secondary outcomes

**Fig. 6:**
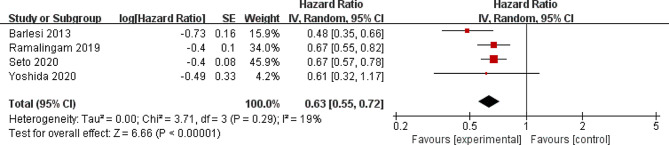
Forest plot for the meta-analysis of progression-free survival

**Fig. 7:**
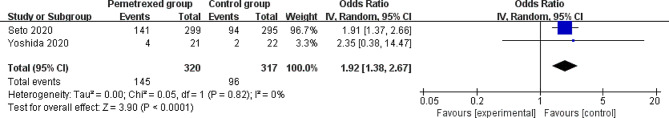
Forest plot for the meta-analysis of progression-free survival rate

**Fig. 8:**
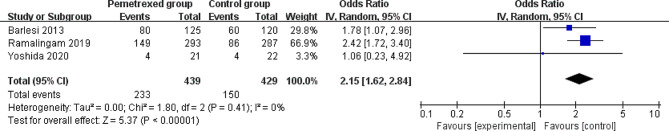
Forest plot for the meta-analysis of grade ≥ 3 adverse events

Compared with control group for NSCLC, pemetrexed addition showed substantially improved progression-free survival (moderate quality, HR = 0.63; 95% CI = 0.55 to 0.72; *P* < 0.00001; Fig. 6) and progression-free survival rate (moderate quality, OR = 1.92; 95% CI = 1.38 to 2.67; *P* < 0.00001; Fig. 7). With regard to the safety, pemetrexed addition resulted in the increase in grade ≥ 3 adverse events (moderate quality, OR = 2.15; 95% CI = 1.62 to 2.84; *P* < 0.00001; Fig. 8).

## Discussion

In the PARAMOUNT trial, pemetrexed supplementation was able to significantly prolong OS and PFS [28, 29]. Pemetrexed plus bevacizumab was significantly associated with improved PFS versus maintenance therapy with single-agent bevacizumab [27, 30]. In contrast, one recent study reported no increase in OS after the treatment with pemetrexed plus bevacizumab (*P* = 0.28) in patients with advanced NSCLC [19].

Considering these inconsistent results, our meta-analysis aimed to confirm the efficacy of pemetrexed plus bevacizumab versus bevacizumab for patients with NSCLC. We included four RCTs and 1467 patients. The results suggested that compared to bevacizumab intervention, pemetrexed plus bevacizumab substantially improved overall survival, survival rate, progression-free survival and progression-free survival rate for patients with NSCLC.

In terms of sensitivity analysis, although there was no significant heterogeneity, several factors may produce some bias. Firstly, the stages of NSCLC were different among the included patients, including metastatic and advanced cancers. Secondly, subgroup histologic types of NSCLC included squamous and non-squamous types, which may have different sensitivity to pemetrexed. Thirdly, the treatment duration of pemetrexed addition varied from 8 months to 63 months, which may affect the efficacy assessment of pemetrexed plus bevacizumab.

With regards to the safety, pemetrexed addition was associated with increased incidence of grade ≥ 3 adverse events for NSCLC patients. The most common adverse events mainly included neutropenia, thrombopenia and anemia, leukopenia. They were generally tolerant after corresponding treatments [18]. The prognosis of NSCLC was poor, especially for metastatic NSCLC [31]. Many novel signatures such as lncRNAs and autophagy-related genes may be able to evaluate the prognosis of cancers [32, 33]. For instance, dual homeoboxes A pseudogene 8 (DUXAP8) was closely related to poor overall survival in several cancers, suggesting its ability to serve as a prognostic biomarker and potential therapeutic target for cancers [34]. As the development of immunohistochemical markers in the subclassification of NSCLC, immunotherapy emerged as an increasingly important option [35, 36].

We should also consider several limitations. Firstly, our analysis was based on only four RCTs and more studies with large patient samples should be conducted to confirm our findings. Secondly, the treatment duration of pemetrexed treatment were different in the included studies, and may lead to some heterogeneity. Thirdly, NSCLC patients with different stages and subgroup histologic types may produce some bias.

## Conclusion

Pemetrexed addition to bevacizumab may improve the treatment efficacy for NSCLC patients.

## Data Availability

Not applicable.
